# Academy of Stomatology

**Published:** 1898-02

**Authors:** 


### ACADEMY OF STOMATOLOGY.

A regular meeting of the Academy of Stomatology of Philadelphia was held November 23, 1897, Dr. Edwin T. Darby in the chair.

A paper on "Arsenical Necrosis" was read by Dr. Otto E. Inglis. (For Dr. Inglis's paper, see page 76.)

DISCUSSION.

*Dr. S. H. Guilford.*—The subject which has been chosen by the essayist is an interesting one. We have all of us had more or less experience with the escape of arsenic upon the gum, though such an accident is not likely to occur where reasonable care has been observed. The fault in such cases may lie in the contributory negligence of the patient. I recollect one case in my early practice where I applied arsenic to two bicuspids in the superior jaw of a young lady, giving instructions to her to carefully maintain in position the coverings of cotton and sandarach varnish which it was then the custom to use. She was to return in a day or two, but I did not see her for a month. Upon examination I found considerable necrosis, and a portion of the septum had to be removed. Our greatest safeguard undoubtedly lies in the careful sealing of the application in the cavity, though I fail to see why zinc phosphate is not as generally applicable for this purpose as facing amalgam.

*Dr. James Truman.*—I was pleased with the essay, and especially that portion of it which dealt with the theory of the action of arsenic upon the tissues. There has been but comparatively little attention paid to the phase of the subject selected by the essayist, and any one who has been in a position to observe knows the carelessness with which frequently students handle this drug. I cannot agree with the essayist in regard to the use of ferric hydrate. In those cases of necrosis in which I have been consulted by students I have generally been able to control the action of the arsenic with dialyzed iron, which is equally a chemical antidote for arsenic and is a more available drug.

If left to itself arsenic acts upon the gum-tissue, penetrating more and more deeply until it becomes inert; then that portion which has been chemically destroyed is thrown off. It is true that even with the use of dialyzed iron some tissue will be sequestered, but I believe it to be only that which is destroyed before the case is taken in hand, and that the action would have been greater had not the antidote been used.

*Dr. H. C. Register.*—The essayist has certainly brought out some nice points in regard to the action of arsenic. A very few cases have occurred in my practice, and in each instance the action upon the gum-tissues has been slight and seemingly controlled by the application of the ordinary antidotes. In applications to the pulp, I have found the action always happier when the hyperæmia of the pulp is first relieved by depletion. Carbolic acid has not seemed to act so well as a menstruum as other and milder medicaments, such as the oil of cinnamon.

According to the experiments of E. Riegal, arsenic does not act upon mucous membranes unless the cuticle is in some way broken or injured. When this has occurred, however, the action is only limited by the power which the given quantity of arsenic has of combining with the tissue. It may be that the use of carbolic acid as a menstruum may effect that injury which is necessary for the action of the arsenic upon the gums.

I have great faith in the ferric hydrate as an antidote, but it should be used in quantity sufficient; it being calculated that twelve times the amount of the arsenic is the quantity necessary to effect neutralization.

*Dr. G. C. Chance.*—A personal experience has led me to believe that there is a great evil practised by druggists in dispensing a preparation of arsenic for relief of toothache. One evening, when suffering from pulpitis, I asked of a nearby druggist a preparation of oil of cloves and morphine acetate. He assured me that he had a preparation containing only those ingredients. Reluctantly I accepted his cure, with the result of increased pain. Upon examination next day by a professional friend necrosis of the gum septum was found. Inquiry elicited the fact that incorporated with the mixture was also a percentage of arsenic. As not only this particular druggist, but several others of whom I have inquired, have wonderful faith in this preparation, I think that we should caution their profession against the evil results likely to come to the patient from the use of arsenic in so careless a manner.

*Dr. H. B. Hickman.*—In November, 1896, a lady came to my office with the inferior left first bicuspid abscessed with fistula of several years' persistence. After removing an amalgam filling and extirpating a dead pulp, I treated in the usual manner with carbolic acid, creosote, etc., but could not close the fistula for more than a few days at a time. After exhausting the list of medicinal agents I filled the pulp canal with aristo-paraffin, forcing it through the fistula until it appeared upon the gum. On the point of the wax I found a small particle of amalgam, and thought I had discovered the cause of the trouble. An unusual amount of aristo-paraffin was required.

As the fistula continued to recur, I dissected the gum back and with a large bur cut out all the necrosed bone lining a pus-cavity of considerable size, into which my paraffin root filling had probably been forced in the first instance. I then smoothed the root. After flooding the bone-cavity with a disinfectant, I dried it as well as possible, filled the root with aristo-paraffin, and drew the lips of the wound together with a stitch. Up to the present time the fistula has not returned. The second bicuspid was lost ten years ago as the result of an arsenical application carelessly secured. Is it possible that the necrosis over the first bicuspid root could have been the result of arsenical action?

*Dr. M. J. Schomberg.*—There is considerable room for scientific thought relative to manifestations of the local action of arsenic on living tissues. In explanation of the manner in which these manifestations, namely, ulceration, death, and necrosis, are brought about, a few facts should, to my mind, be taken into consideration. In the first place, we are all familiar with the preservative property of arsenic, it being used in the preparation of cadavers for preservation. Then again we know of its marked power of penetration. In a few cases where arsenic was taken with suicidal intent into the alimentary canal, post-mortem examination revealed by the test for arsenic that it was present in the brain. Now, granting that the chemical properties of dead tissue which has not undergone putrefaction is precisely the same as living tissue with the exception of vital force, and noting that in the former it preserves and in the latter it destroys, we must come to the same conclusion as the essayist,—viz., the cause of necrosis of the tissue is the stasis within the blood-vessels. Again, this explains why the necrosis is localized, congestion preventing the absorption and distribution of this irritant poison. However, when portions of sequestra are thrown off the arsenic resident in the mass may attack adjacent parts.

*The President.*—We will ask Dr. Inglis to kindly close the discussion.

*Dr. Inglis.*—The first speaker mentioned the relative merits of amalgam and zinc phosphate as coverings for arsenical applications. My own experience with zinc phosphate is that in proportion to the difficulty of placing the covering is it an unmanageable material. If used in a very soft condition it adheres readily to everything except the desired parts, and if mixed stiff it requires an undue amount of pressure. Where the rubber dam can be used, and in situations in which it can be flowed over the application, it is admirable. I was glad Dr. Truman mentioned the carelessness of students in this matter of arsenical applications. I recall one case in which I was consulted by a young graduate who had applied to the exposure in a buccal cavity of a lower molar a quantity of the S. S. White paste equal to twice the size of an ordinary pin-head. This he covered by squeezing temporary stopping directly down upon the application. This was gross carelessness and ignorance, yet this gentleman was a good operator from the manipulative point of view.

It is very difficult to estimate the value of dialyzed iron in cases of arsenical necrosis, inasmuch as the quantity of arsenic escaped varies so greatly, also the time elapsing between the inception of the trouble and the presentation of the patient is a factor which renders it doubtful if dialyzed iron is of more value than mere mechanical removal of the arsenic. There seems to be no case in which the arsenic is not self-limited in action. These two factors—quantity and persistence of application—seem to control the extent of the necrosis.

